# Hypertensive Disorders in Pregnancy Are Associated With Congenital Heart Defects in Offspring: A Systematic Review and Meta-Analysis

**DOI:** 10.3389/fcvm.2022.842878

**Published:** 2022-03-28

**Authors:** Senmao Zhang, Xing Qiu, Tingting Wang, Letao Chen, Jinqi Li, Jingyi Diao, Yihuan Li, Jiabi Qin, Lizhang Chen, Yurong Jiang

**Affiliations:** ^1^Department of Epidemiology and Health Statistics, Xiangya School of Public Health, Central South University, Changsha, China; ^2^Hunan Provincial Key Laboratory of Clinical Epidemiology, Changsha, China; ^3^Xiangya Nursing School of Central South University, Changsha, China; ^4^National Health Commission Key Laboratory for Birth Defect Research and Prevention, Hunan Provincial Maternal and Child Health Care Hospital, Changsha, China; ^5^Department of Obstetrics, Hunan Provincial Maternal and Child Health Care Hospital, Changsha, China

**Keywords:** congenital heart defects, pre-eclampsia, gestational hypertension, chronic hypertension, meta-analysis, hypertensive disorders of pregnancy

## Abstract

**Background:**

Although research indicates an association between hypertensive disorders of pregnancy (HDP) and congenital heart defects (CHDs) in offspring, consistency is still lacking. Therefore, we aimed to synthesize the updated published epidemiologic evidence to estimate the association of maternal HDP with the risk of total CHDs and its phenotypes in offspring.

**Methods:**

A systematic search of Web of Science Database, PubMed, and Embase were searched from inception through April 30, 2021 based on a preprepared protocol, and the reference lists were also manually searched. The combined risk estimates were calculated using either the fixed-effect models or random-effect models. Possible heterogeneity moderators were detected by subgroup, sensitivity analyses, and Galbraith plot.

**Results:**

Twenty-four studies involving 477,839 CHDs cases among 40,394,699 participants were included in our meta-analysis. Mothers who had HDP exposure were significantly associated with an increased risk of total CHDs compared with non-exposure. When maternal HDP exposure was further subdivided into pre-eclampsia (*OR* = 1.79, 95% CI: 1.50–2.13), gestational hypertension (*OR* = 1.16, 95% CI: 1.02–1.31), and chronic hypertension (*OR* = 1.68, 95% CI: 1.49–1.89), a significantly increased risk of total CHDs were still presented. Furthermore, a statistically significant increased association was found between maternal HDP exposure and most CHD phenotypes. Besides, relevant heterogeneity moderators have been identified by subgroup and sensitivity analyses.

**Conclusion:**

Our study suggested that maternal HDP exposure may be associated with an increase in the risk of CHDs in offspring. These findings highlight the need for greater surveillance of pregnant women with HDP exposure to allow early prevention that may be good for reducing the risk of CHDs in offspring.

**Clinical Trial Registration:**

[www.ClinicalTrials.gov], identifier [CRD42021268093].

## Introduction

Congenital heart defects (CHDs), defined as gross structural malformation of the heart or large blood vessels during the fetal period, are the most frequent congenital anomalies in newborns, affecting 8.6–10.3 per 1,000 live births (1–3). Heart defects are a major cause of infant non-infectious morbidity and mortality, which can lead to about 6.0% neonatal and 46% congenital deaths (4, 5). Evidence showed that more than one million children with CHDs are born every year in the world, and more than half of them need surgical intervention within 1-year-old (6, 7). Although the surgical repair of CHD has achieved remarkable success, many interventions are palliative rather than curative (8). Survivors with CHDs are usually at significant risk of cardiovascular complications and neurodevelopmental disorders in the long term (8, 9). At present, the cause of CHDs is largely unknown.

Hypertensive disorders in pregnancy (HDP) are a group of maternal disorders characterized by increased blood pressure during pregnancy, which include gestational hypertension, pre-eclampsia or eclampsia, chronic hypertension, and preeclampsia superimposed on chronic hypertension (10). It was estimated that approximately 5–8% of all pregnant women worldwide were affected by HDP (11). Previous studies suggested that HDP produces an adverse *in utero* environment, which may increase the risk of adverse pregnancy outcomes (for example, low birth weight, neonatal death, intrauterine growth restriction, congenital malformations, and so on) (12, 13). These indicated that mothers with HDP may increase the risk for heart defects in offspring. Despite some published original studies that have evaluated the relationship between HDP and CHDs risk, the findings were still inconsistent (14–18).

Until now, one meta-analysis has been published to discuss this question (19). This study reported a significant association between maternal hypertension and the risk of CHDs in offspring (19). However, this review did insufficient consider the heterogeneity of results when exploring the association of maternal hypertension with CHDs (19), so the risk estimates are not entirely precise and robust. Meanwhile, the association between hypertension and specific CHD phenotypes was limited in terms of sample size, which may cause the bias of results due to the lack of statistical power. Additionally, of note, the previous review did not focus on the associated different types of HDP with the risk of CHDs. Furthermore, given the increasing prevalence of HDP, and partially after the publication of that meta-analysis, several large-scale cohort studies with inconsistent outcomes have been reported, collating the existing evidence of the association of HDP with CHDs is required (14–18).

Therefore, based on the above-mentioned situation, we synthesized the available published studies on the association between maternal HDP and the risk of CHDs in offspring in a systematic review and meta-analysis. The objective of this study is as follows: (i) to review and summarize the epidemiologic evidence on the association of maternal HDP on total CHDs and specific CHD phenotypes in offspring; (ii) to estimate the association of different types of HDP with total CHDs in offspring; and (iii) to identify potential sources of heterogeneity by subgroup, sensitivity analyses, and Galbraith plot.

## Materials and Methods

### Literature Search Strategy

We conducted and reported this study by following the protocol of the Preferred Reporting Items for Systematic Reviews and Meta-analyses (PRISMA) statement (20). Web of Science Database, PubMed, and Embase was searched from inception through April 30, 2021. Search terms associated with HDP and CHDs were combined according to the principles of Boolean logic (using AND, OR, or NOT). For example, (hypertensive disorders in pregnancy OR gestational hypertension OR preeclampsia) AND (congenital heart disease OR congenital heart defect OR congenital heart malformation). The full search strategy is included in Supplementary Table 1. Additionally, the reference lists of relevant studies and reviews were also manually searched. The protocol for this systematic review and meta-analysis was registered on PROSPERO, the international prospective register of systematic reviews (CRD42021268093), and subsequently published.

### Exposure and Outcomes

The exposures of interest were HDP, which was defined as a group of characteristic diseases that occur during the gestational period. HDP was usually divided into four categories: gestational hypertension, preeclampsia/eclampsia, chronic hypertension, and preeclampsia superimposed on chronic hypertension. The definitions of HDP of individual studies were guided by guidelines in place at the time of each study in our study. Traditionally, gestational hypertension was defined as new-onset elevated blood pressure (≥140/90 mmHg) after 20 weeks of gestation, and recovery before 12 weeks of delivery (21, 22). Preeclampsia was defined as hypertension (≥140/90 mmHg) and proteinuria (≥300 mg protein per day) developing after 20 weeks of gestation in women who were previously normotensive (21, 22). Chronic hypertension was defined as increased blood pressure (≥140/90 mmHg) before 20 weeks gestation, but not associated with additional systemic features of preeclampsia (21, 22). Chronic hypertension with superimposed preeclampsia was defined when ≥1 of the systemic features of preeclampsia develops in women with chronic hypertension (21, 22). Additionally, considering the possibility that CHDs could be more strongly associated with some variants of preeclampsia, preeclampsia was further classified as mild or severe preeclampsia according to the disease severity, and early onset preeclampsia (less than 34 weeks of gestation) or late-onset preeclampsia (greater than or equal to 34 weeks) based on the disease onset (23).

The outcomes of interest were CHDs or specific CHD phenotypes including conotruncal defects (CTD), atrial septal defects (ASD), ventricular septal defects (VSD), atrioventricular septal defect (AVSD), tetralogy of fallot (TOF), transposition of the great vessels (TGA), coarctation of the aorta (COA), right ventricular outflow tract obstruction (RVOTO), left ventricular outflow tract obstruction (LVOTO), hypoplastic left heart syndrome (HLHS), and heterotaxia, which were confirmed by surgery and/or diagnosed by ultrasonography.

### Study Selection

Two authors (SMZ and TTW) independently initial screened titles and abstracts of all studies, and then reviewed full texts of relevant studies when necessary. Eligibility criteria for fulfilling in our meta-analysis included the following: (i) cohort or case-control studies; (ii) HDP were the exposure of interest including gestational hypertension, pre-eclampsia or eclampsia, chronic hypertension, and preeclampsia superimposed on chronic hypertension; (iii) CHDs or specific CHD phenotypes (e.g., ASD, VSD, TOF, etc.) were the outcomes of interest; (iv) the association between HDP and CHDs or specific CHD phenotypes were part of the main objective of the study (including studies that investigated other perinatal risk factors in addition to HDP); (v) reported odds ratios (ORs) or relative risks (RRs), with corresponding 95% confidence intervals (CIs) (or provided sufficient information to calculate effect value, such as β coefficient and standard error (se), or complete four grid table data (2 × 2 tables) which is sufficient to calculate their OR value or RR value); (vi) published in English-language. On the contrary, exclusion criteria of studies were as follows: (i) reviews, meta-analysis, case reports, letters, and conference abstracts; (ii) without sufficient or clear data; (iii) duplicate publications (If the same population was studied in more than one study, we included the study with the longest follow-up time or the most information). Additionally, we also excluded some studies that focused on the treatment of HDP or special populations (e.g., very low birth weight preterm infants).

### Data Extraction and Quality Assessment

Two authors (SM.Z and TT.W) independently extracted data and assessed study quality for each included literature using a standardized data collection form. All discrepancies were resolved through discussion with third authors (X.Q) until consensus could be reached. Information extracted included the first author’s, year of publication, recruitment period, geographic region, study design, sample sources, sample sizes, the exposure of interest, the outcome of interest, whether confounding factors were adjusted, and quality assessment. Quality assessment of all included studies was conducted by using the Newcastle-Ottawa Scale (24). With this tool, each study is evaluated from three dimensions: the selection of study groups, the comparability of groups, and the ascertainment of the interest of exposure or outcome for case-control or cohort studies. The Stars awarded for each quality item (a total of 9 items) can be used as a quick visual assessment. A study that received greater than or equal to 7 stars was considered of high methodological quality.

### Statistical Analysis

OR and their corresponding 95% CIs were used as the common measure of association of maternal HDP exposure with the risk of CHDs in offspring. Since CHDs were rare (the prevalence was 8.6–10.3 per 1,000 live births), we considered OR as RRs. Random-effect models were used to calculate the combined risk estimates when there is heterogeneity across studies, otherwise, the fixed-effect model was used (25). The Q statistics (significance level at *P* < 0.10) and *I*^2^ statistic (significance level at *I*^2^ > 50%) were identified as a quantitative measurement index for detecting the heterogeneity between studies (26).

Subgroup analyses were performed according to the following factors: geographic region, sample sources, study design, sample sizes, publication years, types of HDP, whether the confounding factors were adjusted, and quality assessment. Sensitivity analyses were conducted to estimate the effect of removing one or more studies at a time on the overall risk estimate. Meanwhile, a Galbraith plot was also conducted to detect the heterogeneity due to individual studies. Besides, funnel plots and Egger’s test was used to evaluate the publication bias (significance level at *P* < 0.10) (27). Data were analyzed using Review Manager Version 5.3 and Stata version 12.0. Each effect size was weighted by the inverse of its variance. Statistical analyses were performed using two-sided *P*-values, and *P* < 0.05 was viewed as statistically significant unless otherwise specified.

## Results

### Literature Search

**FIGURE 1:**
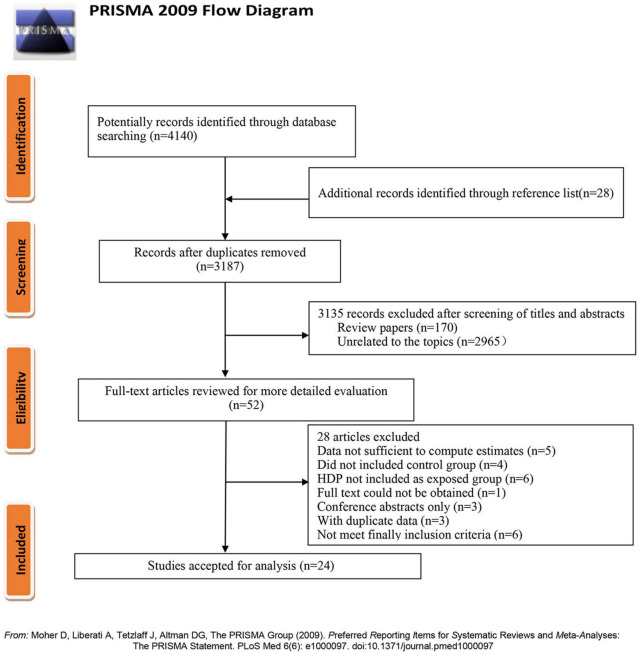
Flow chart of literature selection.

We initially searched 4,140 possibly relevant literature from three databases. The original search engendered 3,187 unique results after excluding duplicate literature. Meanwhile, most articles (*n* = 3,135) were further eliminated after screening titles and abstracts. Then 52 potential articles were carefully reviewed by full-text. Of these, 28 articles were further removed because they did not meet the inclusion criteria. Finally, a total of 24 articles (14–18, 28–46) were included in our meta-analysis. The detailed process is summarized in Figure 1.

### Characteristics of Study

All included literature that was published from 1989 to 2021, with a summary of 477,839 CHDs cases among 40,394,699 participants, are provided in Table 1. Of them, five studies were conducted in Asia, eight in Europe, eight in North America, and the remaining three studies were from South America, Oceania, and Africa. Thirteen studies were based on community populations, while the remaining studies were hospital populations. Seventeen studies belonged to cohort design and seven studies were case-control studies. Among them, most studies (*n* = 15) controlled for possible confounding factors when evaluating the association of maternal HDP with CHDs risk. Fifteen studies had a higher methodological quality; these studies devote most research participants. Additionally, based on the types of HDP, pre-eclampsia was reported in ten studies, gestational hypertension in six studies, chronic hypertension in twelve studies, and preeclampsia superimposed on chronic hypertension in one study.

**TABLE 1:** Characteristic of studies of maternal HDP exposure and risk of congenital heart defects in offspring.

References	Geographic region	Study design	Sample source	Recruitment period	No. of Cases/Controls*	Types of exposure	Outcomes	Whether the confounding factors were adjusted or matched	Quality scores
Stoll et al. (28)	France (European)	Case-control	Hospital	1979–1986	801/801	CH	CHDs/VSD/ASD	Crude	5
Tikkanen and Heinonen (29)	Finland (European)	Case-control	Hospital	1982–1984	573/1055	CH	CHDs	Adjusted	6
Strobino et al. (30)	America (North America)	Case-control	Hospital	1985–1995	796/704	CH	CHDs	Crude	5
Cedergren and Kallen (31)	Sweden (Europe)	Cohort	Population	1992–2001	6346/770355	PE	CHDs	Adjusted	7
Zen et al. (32)	Brazil (South America)	Case-control	Hospital	2005–2006	250/330	CH	CHDs	Crude	5
Mateja et al. (33)	America (North America)	Case-control	Hospital	1996–2005	234/934	CH	CHDs	Adjusted	6
Csáky-Szunyogh et al. (36)	Hungary (Europe)	Case-control	Population	1980–1996	598/38151	CH	CTD	Matched	7
Liu et al. (35)	Canada (North America)	Cohort	Population	2002–2010	23200/ 2278838	CH	CHDs/CTD/VSD/ASD/ AVSD/LVOTO/RVOTO/ Heterotaxia	Adjusted	9
Tabib et al. (34)	Iran (Asia)	Case-control	Hospital	2008–2012	14/155	CH	CHDs	Crude	6
Demirpence et al. (37)	Turkey (Asia)	Cohort	Hospital	2010–2012	66/337	GH	CHD	Adjusted	6
Auger et al. (38)	Canada (North America)	Cohort	Population	1989–2012	1219/1942072	PE	CHD/VSD/ASD/TGA/ TOF/COA/ Heterotaxia	Adjusted	9
Bateman et al. (40)	American (North America)	Cohort	Population	2000–2007	NA/878126	CH	CHDs/CTD/VSD/ ASD/LVOTO/ RVOTO	Adjusted	8
Liu et al. (39)	China (Asia)	Cohort	Population	2009–2011	1817/90796	GH	CHDs	Adjusted	8
Brodwall et al. (42)	Norway (Europe)	Cohort	Population	1994–2009	10691/ 914703	GH/PE	CHDs/AVSD/TGA /TOF/COA/HLHS/ Heterotaxia	Adjusted	8
Chou et al. (14)	China (Asia)	Cohort	Population	2004–2010	23483/ 1387650	CH	CHDs	Adjusted	8
Ruiz et al. (41)	Spain (Europe)	Cohort	Population	2003–2014	279/6314	PE	CHDs	Crude	6
Boyd et al. (15)	Denmark (Europe)	Cohort	Population	1978–2011	17035/ 1972857	GH/PE	CHDs/CTD/VSD/ASD/ AVSD/LVOTO/RVOTO/ Heterotaxia	Adjusted	9
Steurer et al. (43)	American (North America)	Cohort	Population	2007–2012	6903/2989925	CH/GH/PE	CHDs	Crude	7
Weber et al. (44)	American (North America)	Cohort	Population	2007–2011	2041/2209691	CH/GH/PE /PE superimposed on CH	CHDs/TGA/TOF/COA	Adjusted	8
Fantasia et al. (16)	England (Europe)	Cohort	Hospital	2006–2017	206/91407	PE	CHDs	Crude	7
Liu et al. (45)	Australia (Oceania)	Cohort	Hospital	2010–2017	342/73030	HDP	CHDs/TGA/HLHS	Crude	7
Liu et al. (17)	China (Asia)	Cohort	Population	2004–2017	1289/201987	PE	CHDs/VSD/ASD /TGA/TOF/COA/	Adjusted	8
Sanapo et al. (18)	American (North America)	Cohort	Hospital	1997–2012	363116/ 24525889	HDP	CHDs/CTD	Adjusted	7
Yilgwan et al. (46)	Nigeria (Africa)	Cohort	Hospital	2017–2018	45/90	PE	CHDs	Adjusted	6

*CHDs, congenital heart defects; CTD, conotruncal defects; ASD, atrial septal defect; VSD, ventricular septal defect; AVSD, atrioventricular septal defect; TOF, tetralogy of fallout; TGA, D-transposition of the great; COA, coarctation of the aorta; RVOTO, right ventricular outflow tract obstruction; LVOTO, left ventricular outflow tract obstruction; HLHS, hypoplastic left heart syndrome; HPD, hypertensive disorders of pregnancy; PE, pre-eclampsia; GH, gestational hypertension; CH, chronic hypertension; NA, not available.*

*Number of participants with heart defects/total participants are listed when the study was not a case–control study.

### Maternal Hypertensive Disorders of Pregnancy and Risk of Congenital Heart Defects in Offspring

**FIGURE 2:**
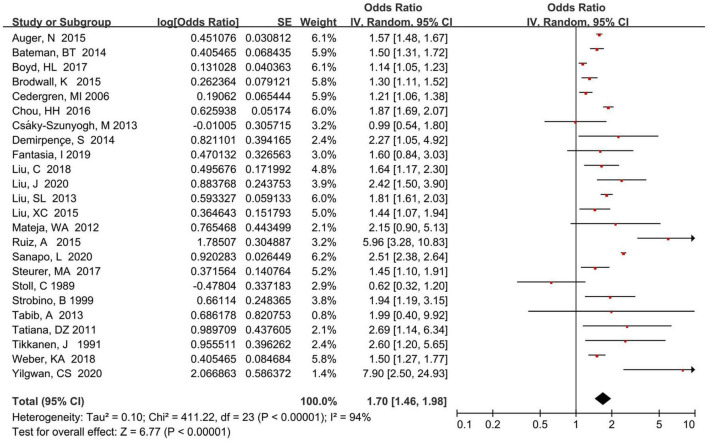
Forest plot of maternal HDP exposure and risk of total CHDs in offspring.

Figure 2 displays the pooled risk estimates between maternal HDP and the development risk of total CHDs in offspring according to random-effect models Overall, mothers who had HDP exposure had a significantly increased risk of total CHDs (*OR* = 1.70, 95% CI: 1.46–1.98; *P* < 0.001) compared with those without HDP exposure. However, substantial heterogeneity was found in the present study (*P* < 0.001; *I*^2^ = 94.0%).

**FIGURE 3:**
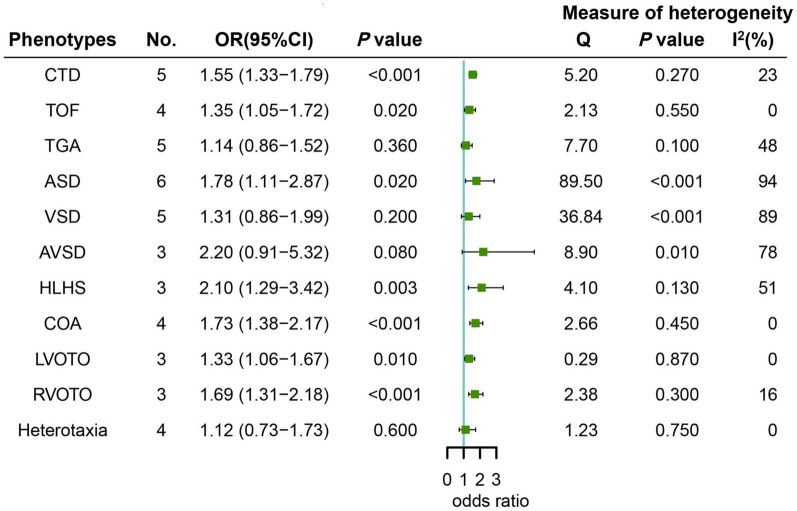
Forest plot of maternal HDP exposure and risk of most CHD phenotypes in offspring.

The results of pooled estimates between maternal HDP and risk of CHD phenotypes are summarized in Figure 3 and detailed forest plots are provided in Supplementary Figures 1–3. Our results indicated that mothers with HDP exposure had a significantly higher risk of most CHD phenotypes including CTD (*OR* = 1.55, 95% CI: 1.33–1.79; *P* < 0.001), TOF (*OR* = 1.35, 95% CI: 1.05–1.72; *P* = 0.020), ASD (*OR* = 1.78, 95% CI: 1.11–2.87; *P* = 0.020),HLHS (*OR* = 2.10, 95% CI: 1.29–3.42; *P* = 0.003), COA (*OR* = 1.73, 95% CI: 1.38–2.17; *P* < 0.001),LVOTO (*OR* = 1.33, 95% CI: 1.06–1.67; *P* = 0.010),and RVOTO (*OR* = 1.69, 95% CI: 1.31–2.18; *P* < 0.001). Nevertheless, there was no substantial heterogeneity except for ASD (*P* < 0.001; *I*^2^ = 94.0%). Additionally, our results indicated that mothers with maternal HDP was not significantly associated with the risk of TGA (*OR* = 1.14, 95% CI: 0.86–1.52; *P* = 0.360), VSD (*OR* = 1.31, 95% CI: 0.86–1.99; *P* = 0.200), AVSD (*OR* = 2.20, 95% CI: 0.91–5.32; *P* = 0.080), and Heterotaxia (*OR* = 1.12, 95% CI: 0.73–1.73; *P* = 0.600). Nevertheless, obvious heterogeneity was observed for several above-mentioned phenotypes except for TGA (*P* = 0.100; *I*^2^ = 48.0%) and Heterotaxia (*P* = 0.750; *I*^2^ = 0.0%).

### Subgroup Analysis

**FIGURE 4:**
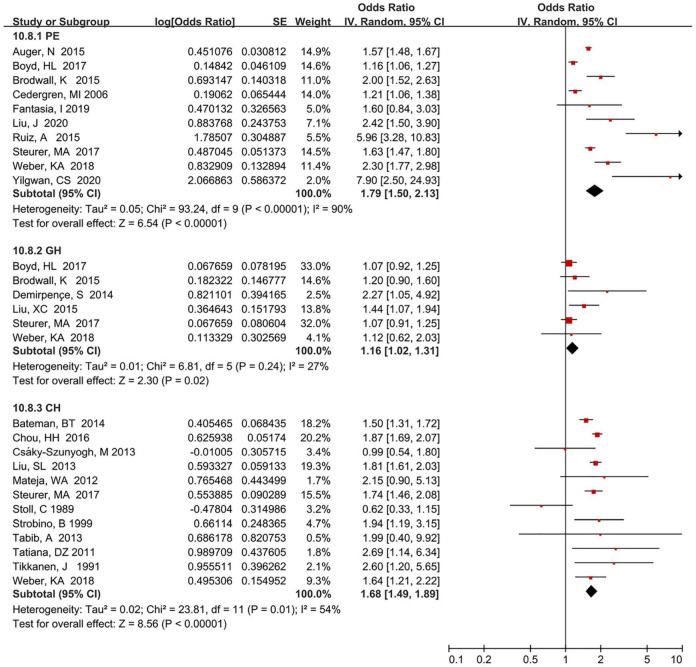
Forest plot of different types of HDP exposure and risk of total CHDs in offspring.

Results of subgroup analyses between maternal HDP and risk of total CHDs were outlined in Table 2. In general, a significantly increased risk of CHDs was found in most subgroups. Subgroup analyses suggested that different types of HDP (test for subgroup difference [TSD]: *I*^2^ = 90.0%), geographic region (TSD: *I*^2^ = 61.8%), sample sources (TSD: *I*^2^ = 63.0%), sample sizes (TSD: *I*^2^ = 61.5%), and quality assessment (TSD: *I*^2^ = 43.4%), were determined as potential heterogeneity moderators. However, a statistically significant difference was only found in different types of HDP (χ^2^ = 29.99; *P* < 0.001) and geographic region (χ^2^ = 13.10; *P* = 0.020), while different sample sources (χ^2^ = 2.70; *P* = 0.100), sample sizes (χ^2^ = 2.60; *P* = 0.110) and quality assessment (χ^2^ = 1.77; *P* = 0.180) were not statistically different. Additionally, subgroup analyses showed that the risk of total CHDs also was significantly increased when data were restricted to specific HDP types (Figure 4), such as pre-eclampsia (*OR* = 1.79, 95% CI:1.50–2.13), gestational hypertension (*OR* = 1.16, 95% CI: 1.02–1.31), and chronic hypertension (*OR* = 1.68, 95% CI: 1.49–1.89). Besides, our results showed that the risk of total CHDs was also significantly increased when restricted to specific preeclampsia types, e.g., mild preeclampsia (*OR* = 1.33, 95% CI: 1.16–1.53), severe preeclampsia (*OR* = 2.31, 95% CI: 2.05–2.60), early onset preeclampsia (*OR* = 4.03, 95% CI: 2.63–6.18), and late-onset preeclampsia (*OR* = 1.27, 95% CI: 1.02–1.57) (Supplementary Figure 4).

**TABLE 2:** Subgroup analysis of association maternal HDP exposure and risk of congenital heart defects in offspring.

Subgroup variables	No. of studies	Pooled OR (95% CI)	Measure of		
			heterogeneity		
			χ^2^	*P*	*I* ^2^
**Geographic region**			13.10*	0.020*	61.8%*
Asia	5	1.84 (1.63-2.07)	4.26	0.370	6.0%
Europe	8	1.38 (1.12-1.71)	39.10	<0.001	82.0%
North America	8	1.74 (1.41-2.15)	170.95	<0.001	96.0%
South America	1	2.69 (1.14-6.34)	NA	NA	NA
Oceania	1	1.64 (1.17-2.30)	NA	NA	NA
Africa	1	7.90 (2.50-24.93)	NA	NA	NA
**Sample sources**			2.70*	0.100*	63.0%*
Community	13	1.54 (1.36-1.75)	116.46	<0.001	90.0%
Hospital	11	1.99 (1.51-2.64)	29.69	0.001	66.0%
**Study design**			0.15*	0.690*	0.0%*
Case-control studies	7	1.58 (1.02-2.44)	14.25	0.030	58.0%
Cohort studies	17	1.73 (1.46-2.05)	395.96	<0.001	96.0%
**Sample sizes**			2.60*	0.110*	61.5%*
≤ 2000	8	2.47 (1.45-4.22)	30.02	<0.001	77.0%
>2000	16	1.56 (1.32-1.85)	375.99	<0.001	96.0%
**Publication years**			1.15*	0.280*	13.2%*
Before 2010	4	1.36 (0.87-2.13)	11.08	0.01	73.0%
2010 or after	20	1.77 (1.50-2.09)	367.72	<0.001	95.0%
**Types of HDP**			29.99*	<0.001*	90.0%*
PE	10	1.79 (1.50-2.13)	93.24	<0.001	90.0%
GH	6	1.16 (1.02-1.31)	6.81	0.240	27.0%
CH	12	1.68 (1.49-1.89)	23.81	0.010	54.0%
PE superimposed on CH	1	2.60 (1.67-4.05)	NA	NA	NA
**Whether the confounding factors were adjusted**			0.00*	0.990*	0.0%*
Adjusted	15	1.70 (1.42-2.04)	379.03	<0.001	96.0%
Unadjusted	9	1.70 (1.19-2.44)	31.51	<0.001	75.0%
**Quality assessment**			1.77*	0.180*	43.4%*
< 7	9	2.23 (1.40-3.55)	35.52	<0.001	78.0%
≥ 7	15	1.59 (1.34-1.89)	375.33	<0.001	96.0%

*HPD, hypertensive disorders of pregnancy; OR, Odd Ratios; 95% CI, confidence interval; PE, pre-eclampsia; GH, gestational hypertension; CH, chronic hypertension; NA, not available.*

*Test for subgroup differences.

### Sensitivity Analysis

**FIGURE 5:**
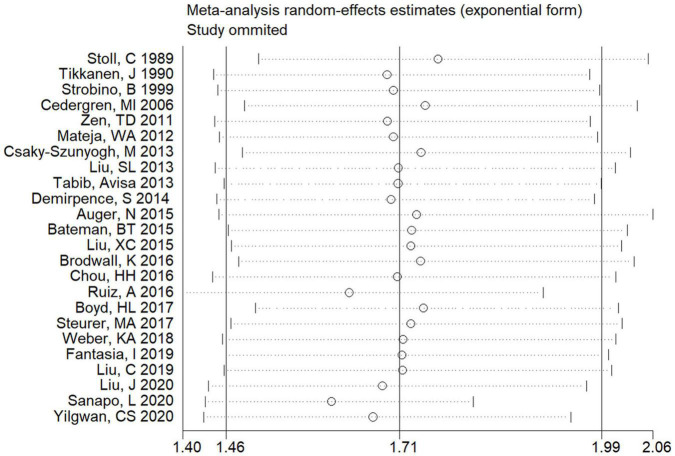
Sensitivity analysis for the association of maternal HDP exposure with risk of overall CHDs in offspring.

Sensitivity analyses were applied to find the possible sources of heterogeneity and to evaluate the effect of various exclusion criteria on pooled risk estimates. Exclusion of poor methodological quality when assessing the association of maternal HDP with the risk of total CHDs did not change the combined results (*OR* = 1.59; 95% CI: 1.34–1.89), but heterogeneity still exists (*P* < 0.001; *I*^2^ = 96.0%). Further exclusion of four old studies (before 2010) also yielded similar results (*OR* = 1.75; 95% CI: 1.48–2.07). Furthermore, removing nine studies that did not control any confounding factors still did not change the combined risk estimate (*OR* = 1.70; 95% CI: 1.42–2.04), but heterogeneity cannot be ignored (*P* < 0.001; *I*^2^ = 96.0%). Besides, removing any single study at a time still did not substantially affect the combined risk estimate (Figure 5).

### Galbraith Plot

**FIGURE 6:**
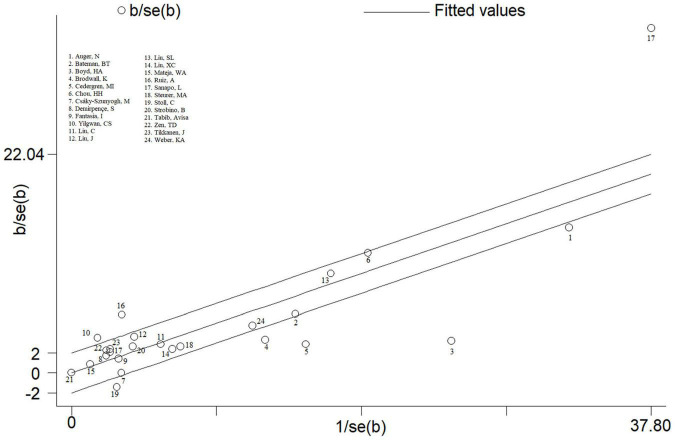
Galbraith plots for maternal HDP exposure and CHDs risk in offspring.

Galbraith plot was performed to find the studies that bring about heterogeneity. Among 24 studies, nine studies were identified after Galbraith plot analysis (Figure 6). After excluding these 9 studies, mothers who had HDP exposure compared with those who did not have HDP exposure still had a significantly increased risk of total CHDs in offspring (*OR* = 1.63, 95% CI: 1.50–1.78; *P* < 0.001). But, it is worth noting that there was an obvious decrease in heterogeneity (*I*^2^ decreased from 94.0 to 15.0%).

### Publication Bias

**FIGURE 7:**
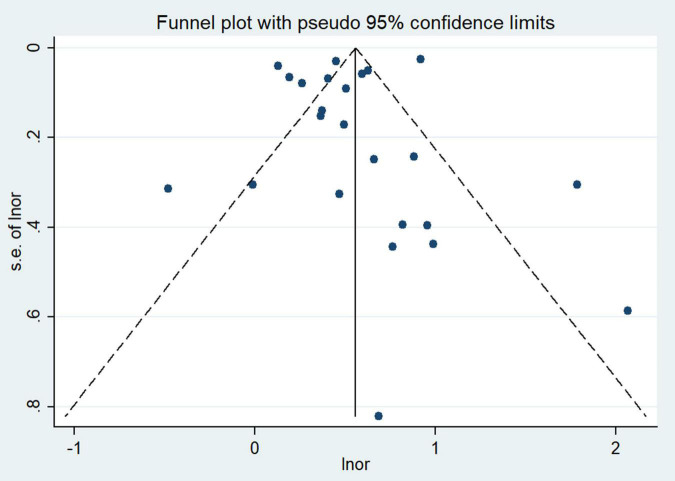
Funnel plot for the association of maternal HDP exposure with risk of overall CHDs in offspring.

Visual inspection of funnel plot showed a mild asymmetry (Figure 7), but Egger’s test showed no significant substantial publication bias between maternal HDP and the risk of total CHDs in offspring (*P* = 0.608).

## Discussion

Our meta-analysis aimed to synthesize the published literature on the association of maternal HDP with the risk of CHDs in offspring. Four principal findings were obtained. First, our study suggested that mothers who had HDP exposure were associated with a 70% increased risk of CHDs in offspring compared with non-exposure. Second, our study indicated that maternal HDP exposure could significantly increase the risk of most CHD phenotypes in offspring including CTD (*OR* = 1.55), TOF (*OR* = 1.35), ASD (*OR* = 1.78), HLHS (*OR* = 2.10), COA (*OR* = 1.73), LVOTO (*OR* = 1.33), and RVOTO (*OR* = 1.69). Third, the results of subgroup analyses indicated that the development risk of total CHDs in offspring had significantly increased when data were confined only to specific HDP types, such as pre-eclampsia (*OR* = 1.79), gestational hypertension (OR = 1.16), and chronic hypertension (*OR* = 1.68). Fourth, our results showed that the risk of total CHDs was also significantly increased when restricted to specific preeclampsia types, e.g., mild preeclampsia (*OR* = 1.33), severe preeclampsia (*OR* = 2.31), early onset preeclampsia (*OR* = 4.03), and late-onset preeclampsia (*OR* = 1.27). These findings indicated that early onset and severe preeclampsia had a higher risk of CHDs than late-onset and mild preeclampsia, respectively, and the increased exposure to the hypertensive disorder could generate more important complications. Besides, our results found that the association of maternal HDP with CHDs still exists after subgroup analyses, sensitivity analyses, and Galbraith plot, which indicated that our findings were credible.

To our knowledge, the potential mechanisms involved in the association of maternal HDP exposure with CHDs risk in offspring remain uncertain. However, several underlying mechanisms have been put forward to try to explain the association of maternal HDP with CHDs risk. The most potential explanation is that maternal HDP can occur in early damage of the vasculogenesis, leading to abnormal development of the placenta (18). Previous studies showed that placental dysplasia may cause alteration in placental genes, oxygen concentration, and protein expression (18). These alterations can lead to altered antioxidant enzyme activity, increased apoptosis, reduction of angiogenic factors, the abnormal balance of growth factors, hypoxic stress, and ultimately to lead to the abnormal development of the fetal cardiovascular system (15, 42, 47). For example, animal studies demonstrated that the blockage of vascular endothelial growth factor (VEGF) receptors resulted in a functional and structural defect in heart valve development, indicating that these receptors are involved in heart valve formation (48, 49). Moreover, animal studies also corroborated that hypoxic stress is associated with abnormal proteins activation and signaling, which may increase the risk of CHD (50). Meanwhile, persisting elevated blood pressure levels may affect cardiovascular health through a variety of mechanisms, including increased afterload, myocardial ischemia, myocardial fibrosis, and mechanical damage on the aortic valve. These alterations may induce abnormally high stress on aortic leaflets, turbulent flow, endothelial injury, and subsequent progression toward alteration in aortic valve morphology, finally causing aortic regurgitation, stenosis, and aortic dilatation, suggesting that persisting hypertension are closely related to cardiac structural abnormalities (51).

Additionally, there are a few possible explanations for the association of maternal HDP with CHDs risk in offspring. On the one hand, evidence indicates that maternal HDP exposure can lead to compromised blood flow to the developing fetus. Decreasing blood flow to the uterus during pregnancy could cause fetal intracardiac blood flow alterations and cell death, thus affecting normal heart development in the fetus (40, 52). On the other hand, although the heart develops were earlier than the period of pre-eclampsia from the theory of primitive embryo development [in embryology, the period of organogenesis starts 3 weeks and ends 8 weeks (42)], researchers have proposed that pathophysiologic changes in preeclampsia begin well before 20 weeks. Recently study has detected imbalances in angiogenic biomarkers as early as the first trimester in women who later developed preeclampsia (38). Among them, overexpression of the antiangiogenic biomarkers soluble endoglin (sEng) and soluble fms-like tyrosine kinase 1 (sFlt-1) associated with angiogenic placental growth factor and vascular endothelial growth factor begins at the start of pregnancy (38, 53). Meanwhile, a researcher found that a fetal imbalance toward anti-angiogenesis at the trophoblast stage could result in abnormal heart formation (38). Therefore, we can speculate that pre-eclampsia may already have clinical changes in early pregnancy, which may affect the development of the heart and lead to an increased risk of CHDs. Additionally, research showed that gestational hypertension may be in the spectrum of pre-eclampsia and was included in the definition of mild preeclampsia (54). Thus, the effect of pre-eclampsia on CHD may be consistent with that of gestational hypertension on CHD. Furthermore, findings regarding the association of maternal HDP with CHDs subtypes are varied in our study. Despite our study suggesting that maternal HDP may increase the risk for most CHDs, the mechanisms are almost unknown. We think that the differences between HDP and specific CHDs phenotypes could be explained by the fact that the pathophysiology of CHDs is multifactorial, involving genetic and environmental factors.

This systematic review had several strengths compared to the existing meta-analysis. First, as far as we know, our study was the first meta-analysis to independently estimate the association of pre-eclampsia, gestational hypertension, and chronic hypertension with the risk of CHDs. Our results indicated that the risk of total CHDs in offspring had significantly increased when data were restricted to pre-eclampsia (*OR* = 1.79), gestational hypertension (*OR* = 1.16), and chronic hypertension (*OR* = 1.68). Second, compared with the previous review (19), our study supplied up-to-date evidence on this topic and involved a large number of studies that not only included America and Europe populations, but also Asia, Oceania, and Africa populations. With the accumulation of evidence and the expansion of sample size and population, we have enhanced our statistical power to provide generalizable risk estimates. Third, although the previous meta-analysis also estimates the association of maternal hypertension with specific CHDs phenotypes (19), the number of studies included in the analysis is limited, including only 2–3 studies. Our study further enlarged the sample size in our analysis, which may contribute to improving the accuracy and reliability of risk estimates. Therefore, different from the results of the previous study (19), our results suggested a significant association of maternal HDP with most CHD phenotypes, which not only included CTD and ASD, but also TOF, HLHS, COA, LVOTO, and RVOTO. Besides, our study also fully considered the effect of heterogeneity and the most relevant heterogeneity moderators have been identified by subgroup analysis.

However, several limitations should be noted in our study. Firstly, in our review, results were limited to English-language literature, which may lead to possible language bias. Moreover, potential publication bias with under-publication of negative results may influence the findings, and it cannot be ignored. Secondly, substantial heterogeneity was found in the our study. This is not surprising, given the different study populations and methodologies. Based on the results of subgroup analyses, we found that types of HDP and geographic region were the potential source of heterogeneity. The possible explanation was that the risk of CHDs and HDP were not consistent in different geographical regions due to differences in race, medical level, and health awareness. For example, in some developing countries (e.g., Africa and Asia), there are still many pregnant women who do not receive adequate antenatal and pregnancy care because of the limited medical service, and the incidence and mortality of HDP were relatively high in these countries. Moreover, the health awareness of pregnant women in these developing countries is relatively weak, which may lead to the diagnosis delay of HDP, and result in a lack of effective early clinical interventions for HDP. Besides, since the definition of HDP may change over time, bias on outcome detection is difficult to avoid, which may increase clinical heterogeneity between studies. Nevertheless, we should still view the results with caution as the heterogeneity cannot be ignored. Thirdly, both case-control and cohort studies were selected in our study. Case-control studies are widely acknowledged to be prone to recall and selection biases, which restrict the strength and quality of evidence. Although the exposure history of maternal HDP was confirmed through medical records and/or physician-diagnosed self-reporting, the potential bias was unavoidable.

Fourth, considering that other subtypes of HDP were mostly diagnosed after organogenesis in addition to chronic hypertension, it is difficult to establish a causal link between pre-eclampsia or gestational hypertension and CHDs based on existing studies. Therefore, our study was mainly used to evaluate the association of maternal pre-eclampsia or gestational hypertension with CHDs in offspring, and could not explain the causal relationship. Fifth, of note, although our results found that early onset and severe preeclampsia had a higher risk of CHDs than late-onset and mild preeclampsia, respectively, the number of studies included in the analysis is limited, only three and four studies. In the future, more studies was needed to further explore the potential association between different types of preeclampsia and the risk of CHDs, which may contribute to improving the accuracy and reliability of risk estimates. Sixth, it is difficult to accurately evaluate the effect of the management of these disorders or the use of antihypertensive treatment on the occurrence of complications because of the limited information from original studies. Last but not least, the potential limitations including these studies (studies that were old or poor consideration of confounding factors) are inevitable. Although most of the included studies have tried to control for some potential confounding factors and restricting the analysis to studies that have controlled confounding factors did not substantially change the pooled risk estimate, the possible influences of confounding factors might not be completely omitted. Therefore, further studies with adequate adjustment of potential confounding factors are still warranted to clarify the association of HDP with CHDs in offspring.

## Conclusion

In conclusion, our findings have two implications significant for the public health of pregnant women. First, our study indicated maternal HDP exposure including pre-eclampsia, gestational hypertension, and chronic hypertension may be associated with an increase in the risk of CHDs. Second, our findings showed that maternal HDP exposure was significantly associated with the risk of most CHD phenotypes in offspring, such as CTD, ASD, and so on. These findings emphasize that maternal HDP exposure could seriously endanger the health of their babies, and lead to an increased risk of death in offspring. Therefore, for pregnancies, especially pregnant women at high risk of HDP, clinical workers should form a coalition of obstetricians, pediatricians, sonographers, and specialists in pregnancy-related hypertensive disease to conduct dynamic monitoring of these pregnant women, and increase cardiac developmental early screening of HDP-exposed infants in order to early intervention, so as to avoid missing the best treatment time and reduce the risk of death in children with CHDs. However, considering that the mechanisms involved in the association between maternal HDP and CHDs in offspring are unclear, further studies are essential to explore the potential mechanisms and find probable interventions.

## Data Availability Statement

The original contributions presented in the study are included in the article/Supplementary Material, further inquiries can be directed to the corresponding authors.

## Author Contributions

SZ, LZC, and YJ made substantial contributions concerning: conception and design, acquisition of data, analysis and interpretation of data, drafting the article and revising it critically for important intellectual content, and final approval of the version to be published. XQ, TW, JL, JD, YL, LTC, and JQ made substantial contributions concerning: analysis and interpretation of data, drafting the article and revising it critically for important intellectual content, and final approval of the version to be published. LZC and YJ was the guarantor for the study. All authors contributed to the article and approved the submitted version.

## Conflict of Interest

The authors declare that the research was conducted in the absence of any commercial or financial relationships that could be construed as a potential conflict of interest.

## Publisher’s Note

All claims expressed in this article are solely those of the authors and do not necessarily represent those of their affiliated organizations, or those of the publisher, the editors and the reviewers. Any product that may be evaluated in this article, or claim that may be made by its manufacturer, is not guaranteed or endorsed by the publisher.
