# Supplementary material for: Hypertensive Disorders in Pregnancy Are Associated With Congenital Heart Defects in Offspring: A Systematic Review and Meta-Analysis
Source: Front Cardiovasc Med. 2022 Mar 28;9:842878. doi: 10.3389/fcvm.2022.842878 (PMC8995565; doi:10.3389/fcvm.2022.842878)
Supplement: Supplementary file 1 [file Data_Sheet_1.pdf]

Supplementary Material

Supplemental Table 1. Search Strategy for Each Electronic Database

1	hypertensive disorders in pregnancy
2	gestational hypertension
3	pregnancy hypertension
4	pregnancy-induced hypertension
5	pregnancy induced hypertension
6	preeclampsia
7	pre-eclampsia
8	eclampsia
9	chronic hypertension complicated with pre-eclampsia
10	chronic hypertension
11	preeclampsia superimposed on preexisting hypertension
12	hypertension
13	# 1 or # 2 or # 3 or # 4 or # 5 or # 6 or # 7 or # 8 or # 9 or # 10 or # 11 or # 12
14	congenital heart disease
15	congenital heart defect
16	congenital heart malformation
17	congenital heart anomalies
18	congenital cardiac disease
19	congenital cardiac defect
20	congenital cardiac malformation
21	congenital cardiac anomalies
22	cardiovascular malformation
23	congenital cardiovascular disease
24	cardiovascular defect
25	cardiovascular anomalies
26	# 14 or # 15 or # 16 or # 17 or # 18 or # 19 or # 20 or # 21 or # 22 or # 23 or # 24 or # 25 or #26
27	case-control study
28	cohort study
29	prospective study
30	longitudinal study
31	follow-up study
32	# 27 or # 28 or # 29 or # 30 or # 31
33	#13 and #26 and #32

Study or Subgroup	log[Odds Ratio]	SE	Weight	Odds Ratio IV, Fixed, 95% CI
**12.1.1 CTD**				
Bateman, BT 2014	-0.10536	0.383693	3.9%	0.90 [0.42, 1.91]
Boyd, HL 2017	0.425268	0.113011	44.6%	1.53 [1.23, 1.91]
Csáky-Szunyogh, M 2013	-0.01005	0.305715	6.1%	0.99 [0.54, 1.80]
Liu, SL 2013	0.536493	0.184157	16.8%	1.71 [1.19, 2.45]
Sanapo, L 2020	0.559616	0.140834	28.7%	1.75 [1.33, 2.31]
**Subtotal (95% CI)**			**100.0%**	**1.55 [1.33, 1.79]**
Heterogeneity: Chi² = 5.20, df = 4 (P = 0.27); I² = 23%				
Test for overall effect: Z = 5.77 (P < 0.00001)				
**12.1.2 TOF**				
Auger, N 2015	0.512824	0.204837	37.4%	1.67 [1.12, 2.50]
Brodwall, K 2015	-0.10536	0.757759	2.7%	0.90 [0.20, 3.97]
Liu, J 2020	0.429182	0.560461	5.0%	1.54 [0.51, 4.61]
Weber, KA 2018	0.157004	0.169116	54.9%	1.17 [0.84, 1.63]
**Subtotal (95% CI)**			**100.0%**	**1.35 [1.05, 1.72]**
Heterogeneity: Chi² = 2.13, df = 3 (P = 0.55); I² = 0%				
Test for overall effect: Z = 2.37 (P = 0.02)				
**12.1.3 TGA**				
Auger, N 2015	-0.46204	0.335632	18.7%	0.63 [0.33, 1.22]
Brodwall, K 2015	-0.10536	0.587394	6.1%	0.90 [0.28, 2.85]
Liu, C 2018	0.311188	0.431227	11.3%	1.37 [0.59, 3.18]
Liu, J 2020	2.530517	1.224486	1.4%	12.56 [1.14, 138.44]
Weber, KA 2018	0.24686	0.18389	62.4%	1.28 [0.89, 1.84]
**Subtotal (95% CI)**			**100.0%**	**1.14 [0.86, 1.52]**
Heterogeneity: Chi² = 7.70, df = 4 (P = 0.10); I² = 48%				
Test for overall effect: Z = 0.91 (P = 0.36)				

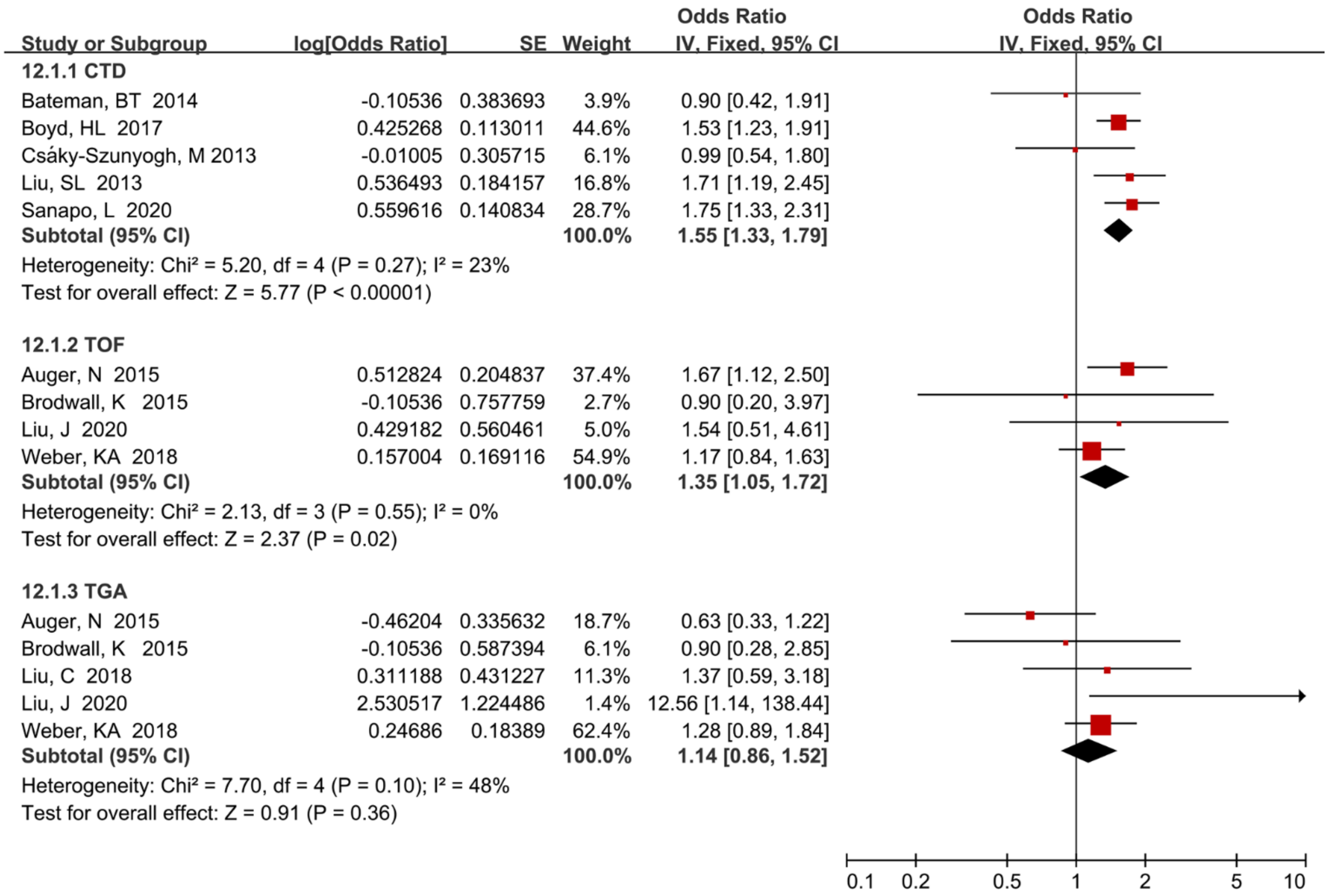

Supplemental Figure 1. Forest plot of maternal HDP exposure and risk of CTD, TOF, and TGA in offspring

Study or Subgroup	log[Odds Ratio]	SE	Weight	Odds Ratio IV, Random, 95% CI
**12.3.1 ASD**				
Auger, N 2015	0.647103	0.049443	19.1%	1.91 [1.73, 2.10]
Bateman, BT 2014	0.262364	0.190616	17.2%	1.30 [0.89, 1.89]
Boyd, HL 2017	-0.09431	0.127422	18.3%	0.91 [0.71, 1.17]
Liu, J 2020	1.879465	0.229564	16.4%	6.55 [4.18, 10.27]
Liu, SL 2013	1.137833	0.131158	18.3%	3.12 [2.41, 4.03]
Stoll, C 1989	-0.84397	0.498673	10.6%	0.43 [0.16, 1.14]
**Subtotal (95% CI)**			**100.0%**	**1.78 [1.11, 2.87]**
Heterogeneity: Tau² = 0.31; Chi² = 89.50, df = 5 (P < 0.00001); I² = 94%				
Test for overall effect: Z = 2.38 (P = 0.02)				
**12.3.2 VSD**				
Bateman, BT 2014	0.09531	0.160359	20.9%	1.10 [0.80, 1.51]
Boyd, HL 2017	0.058269	0.083802	22.9%	1.06 [0.90, 1.25]
Liu, J 2020	1.068153	0.163231	20.8%	2.91 [2.11, 4.01]
Liu, SL 2013	0.41871	0.16143	20.9%	1.52 [1.11, 2.09]
Stoll, C 1989	-0.4943	0.351581	14.4%	0.61 [0.31, 1.22]
**Subtotal (95% CI)**			**100.0%**	**1.31 [0.86, 1.99]**
Heterogeneity: Tau² = 0.19; Chi² = 36.84, df = 4 (P < 0.00001); I² = 89%				
Test for overall effect: Z = 1.28 (P = 0.20)				
**12.3.3 AVSD**				
Bateman, BT 2014	1.589235	0.319582	35.8%	4.90 [2.62, 9.17]
Boyd, HL 2017	-0.08338	0.506905	28.1%	0.92 [0.34, 2.48]
Liu, SL 2013	0.678034	0.312328	36.1%	1.97 [1.07, 3.63]
**Subtotal (95% CI)**			**100.0%**	**2.20 [0.91, 5.32]**
Heterogeneity: Tau² = 0.46; Chi² = 8.90, df = 2 (P = 0.01); I² = 78%				
Test for overall effect: Z = 1.76 (P = 0.08)				
**12.3.4 HLHS**				
Brodwall, K 2015	0.470004	0.587394	14.2%	1.60 [0.51, 5.06]
Liu, C 2018	1.133168	0.254302	39.5%	3.11 [1.89, 5.11]
Weber, KA 2018	0.48858	0.204519	46.3%	1.63 [1.09, 2.43]
**Subtotal (95% CI)**			**100.0%**	**2.10 [1.29, 3.42]**
Heterogeneity: Tau² = 0.09; Chi² = 4.10, df = 2 (P = 0.13); I² = 51%				
Test for overall effect: Z = 2.98 (P = 0.003)				

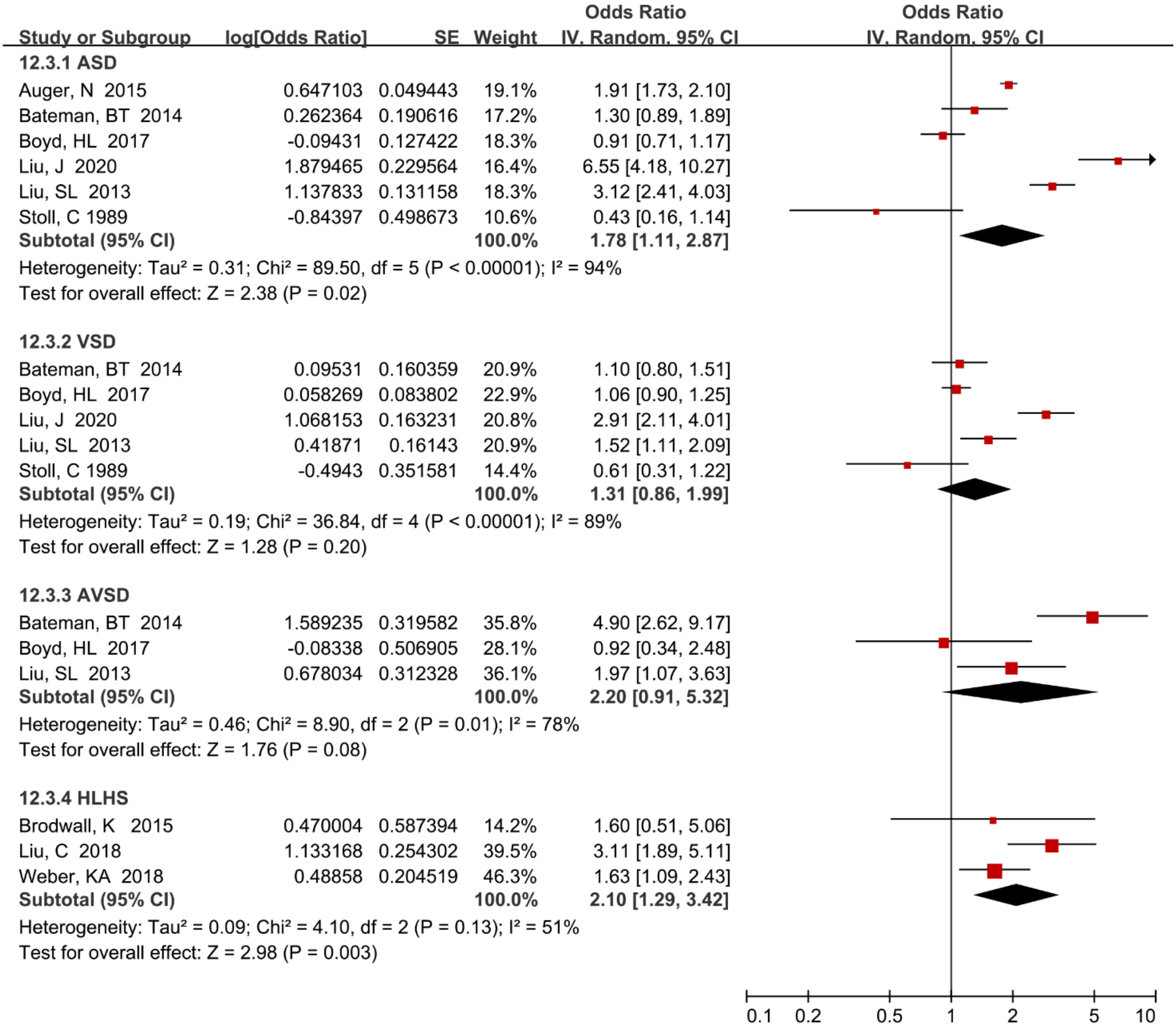

Supplemental Figure 2. Forest plot of maternal HDP exposure and risk of ASD, VSD, AVSD, and HLHS in offspring

Study or Subgroup	log[Odds Ratio]	SE	Weight	Odds Ratio IV, Fixed, 95% CI
**12.2.1 COA**				
Auger, N 2015	0.29267	0.230729	25.4%	1.34 [0.85, 2.11]
Brodwall, K 2015	0.916291	0.465446	6.2%	2.50 [1.00, 6.22]
Liu, J 2020	0.04879	0.726335	2.6%	1.05 [0.25, 4.36]
Weber, KA 2018	0.631272	0.143272	65.8%	1.88 [1.42, 2.49]
**Subtotal (95% CI)**			**100.0%**	**1.73 [1.38, 2.17]**
Heterogeneity: Chi² = 2.66, df = 3 (P = 0.45); I² = 0%				
Test for overall effect: Z = 4.72 (P < 0.00001)				
**12.2.2 LVOTO**				
Bateman, BT 2014	0.09531	0.37796	9.3%	1.10 [0.52, 2.31]
Boyd, HL 2017	0.307485	0.126433	83.1%	1.36 [1.06, 1.74]
Liu, SL 2013	0.307485	0.418111	7.6%	1.36 [0.60, 3.09]
**Subtotal (95% CI)**			**100.0%**	**1.33 [1.06, 1.67]**
Heterogeneity: Chi² = 0.29, df = 2 (P = 0.87); I² = 0%				
Test for overall effect: Z = 2.50 (P = 0.01)				
**12.2.3 RVOTO**				
Bateman, BT 2014	0.262364	0.55333	5.6%	1.30 [0.44, 3.85]
Boyd, HL 2017	0.476234	0.140961	85.7%	1.61 [1.22, 2.12]
Liu, SL 2013	1.153732	0.440332	8.8%	3.17 [1.34, 7.51]
**Subtotal (95% CI)**			**100.0%**	**1.69 [1.31, 2.18]**
Heterogeneity: Chi² = 2.38, df = 2 (P = 0.30); I² = 16%				
Test for overall effect: Z = 4.02 (P < 0.0001)				
**12.2.4 Heterotaxia**				
Auger, N 2015	0.058269	0.345242	40.4%	1.06 [0.54, 2.09]
Boyd, HL 2017	-0.12783	0.411876	28.4%	0.88 [0.39, 1.97]
Brodwall, K 2015	-0.10536	1.064895	4.2%	0.90 [0.11, 7.26]
Liu, SL 2013	0.494696	0.422342	27.0%	1.64 [0.72, 3.75]
**Subtotal (95% CI)**			**100.0%**	**1.12 [0.73, 1.73]**
Heterogeneity: Chi² = 1.23, df = 3 (P = 0.75); I² = 0%				
Test for overall effect: Z = 0.53 (P = 0.60)				

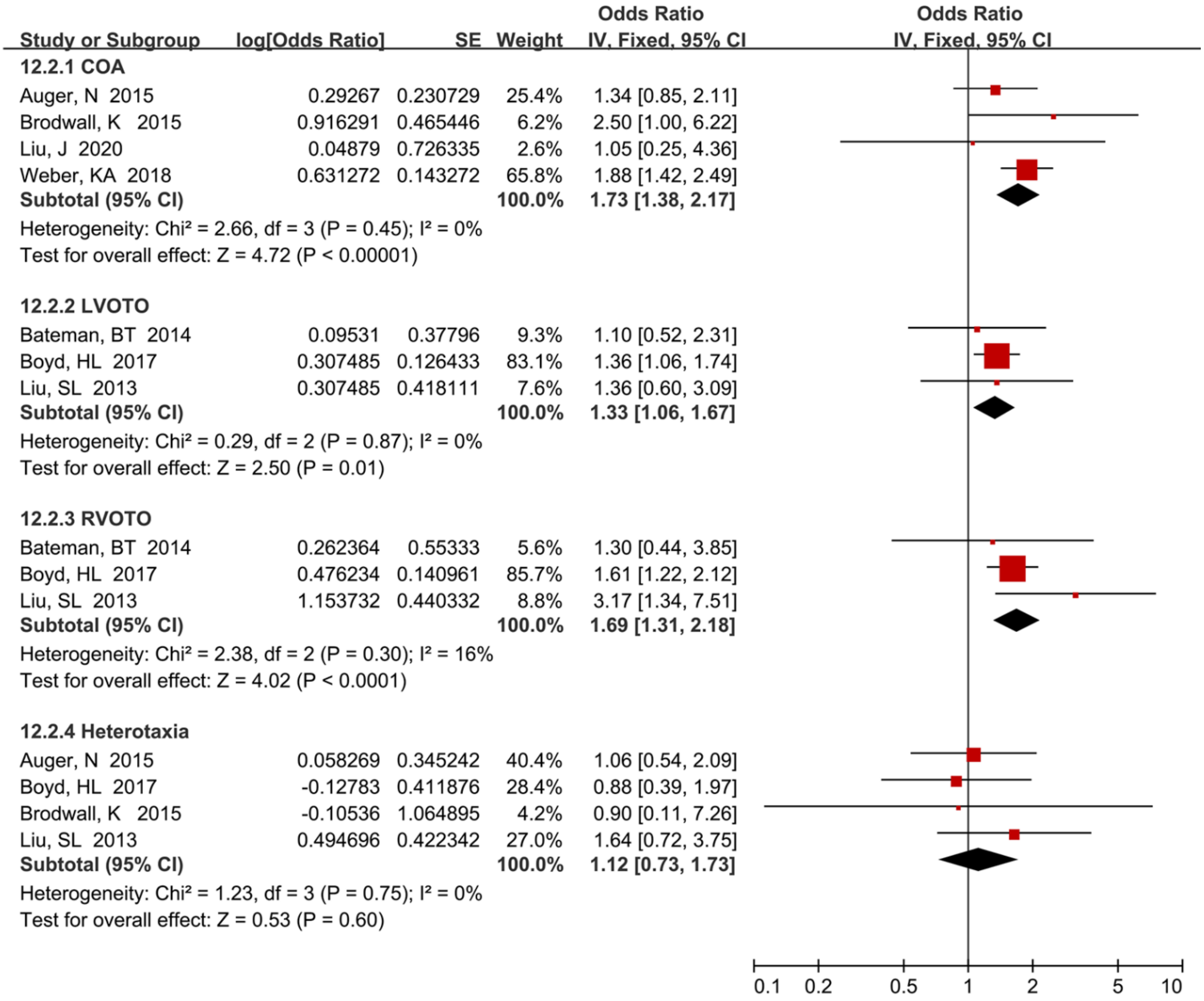

Supplemental Figure 3. Forest plot of maternal HDP exposure and risk of COA, LVOTO, RVOTO, and Heterotaxia in offspring

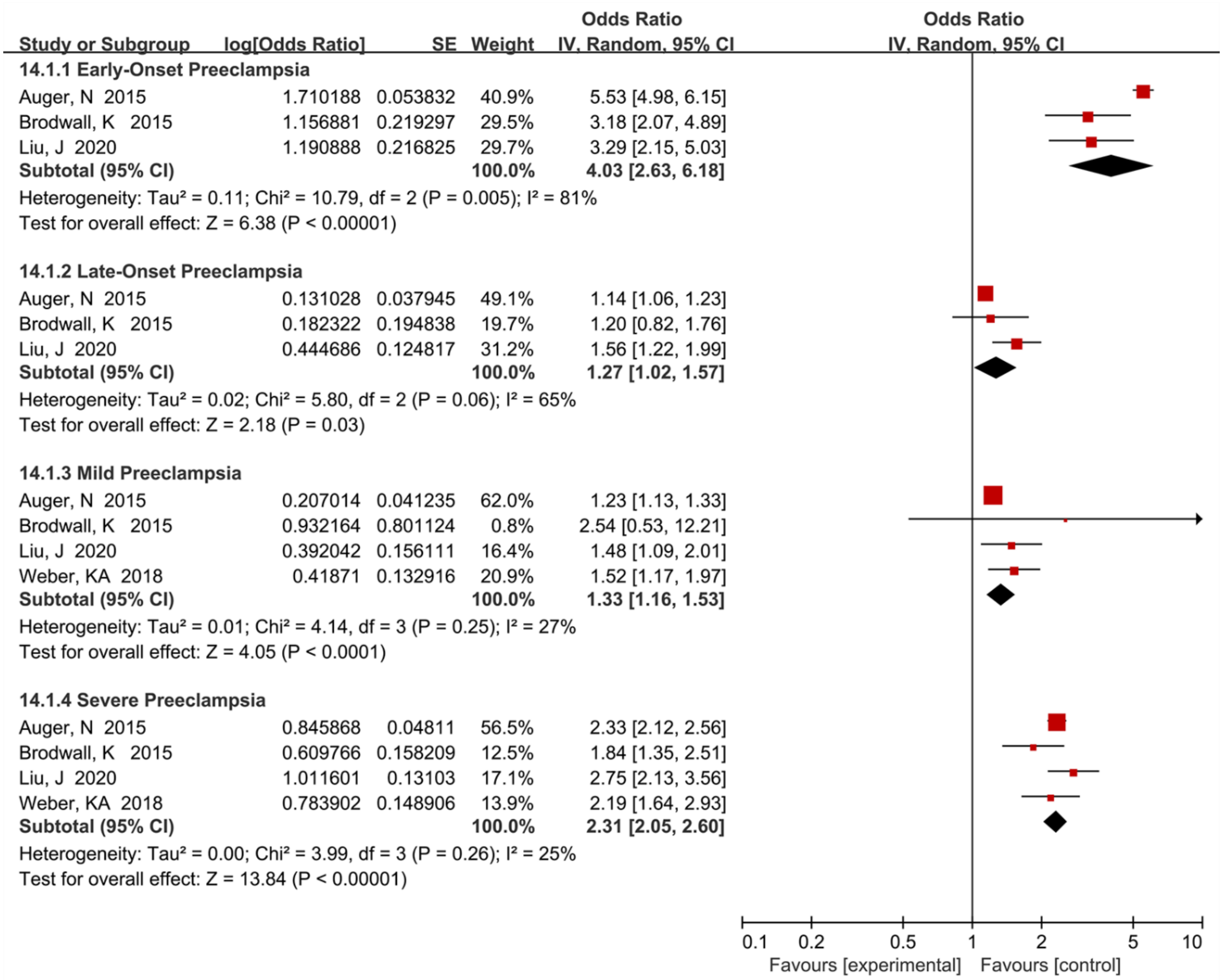

Supplemental Figure 4. Forest plot of specific preeclampsia types and the risk of total CHDs in offspring
